# Supplementary material for: A transparent hybrid metal halide glassy scintillation screen for high-resolution fast neutron radiography
Source: Nat Commun. 2025 Jul 5;16:6215. doi: 10.1038/s41467-025-61503-9 (PMC12228732; doi:10.1038/s41467-025-61503-9)
Supplement: Supplementary file 1 — Supplementary Information [file 41467_2025_61503_MOESM1_ESM.pdf]

Supplementary Information

A transparent hybrid metal halide glassy scintillation screen for high-resolution fast neutron radiography

Zi'an Zhou[1,2], Jinxiao Zheng[1✉], Shihao Ruan[3], Guichu Yue[4], Tiao Feng[1,2], Yini An[1,2], Meimei Wu[3], Nü Wang[5], Shuyun Zhou[1], Linfeng He[3✉], Chenghua Sun[1✉]

[1]Key Laboratory of Photochemical Conversion and Optoelectronic Materials, Technical Institute of Physics and Chemistry, Chinese Academy of Sciences, Beijing,100190, China.

[2]University of Chinese Academy of Sciences, Beijing, 100049, China.

[3]China Institute of Atomic Energy, Beijing, 102413, China.

[4]College of Chemical Engineering, Inner Mongolia University of Technology, Hohhot, 010051, China.

[5]School of Chemistry, Beihang University, Beijing, 100191, China.

✉e-mail: zhengjinxiao@mail.ipc.ac.cn, hlf1212@sina.com, sunchenghua@mail.ipc.ac.cn.

Supplementary Table 1. Comparison of fast neutron scintillator parameters. Density, hydrogen density, fast neutron reaction cross-section and fast neutron stopping power of hybrid metal halide scintillators, ZnS (Ag): PP scintillators, and other commonly used scintillators.

Compound*	Density (g cm^{-3})	Hydrogen density (kg m^{-3})	Elastic scattering cross-section (μ, cm^{-1})	Fast neutron stopping power (Scattered fast neutrons in 1 mm of host) (φ, %)
$(BTPP)_2MnBr_4$	1.454	68.92	0.2698	2.662
$(HTPP)_2MnBr_4$	1.373	75.10	0.2817	2.777
$(DTPP)_2MnBr_4$	1.277	82.61	0.2963	2.919
$(CTPP)_2MnBr_4$	1.223	87.09	0.3052	3.006
$(BTPP)_{1.8}(HTPP)_{0.2}MnBr_4$	1.440	69.40	0.2703	2.667
$(BTPP)_{1.8}(DTPP)_{0.2}MnBr_4$	1.423	70.39	0.2719	2.682
$(BTPP)_{1.8}(CTPP)_{0.2}MnBr_4$	1.407	70.99	0.2726	2.689
ZnS (Ag): PP	1.900	136.9	0.4556	4.454
Stilbene	1.010	67.33	0.2690	2.654
Anthracene	1.280	71.91	0.3100	3.052

* $BTPP^+ = C_{22}H_{24}P^+$; $HTPP^+ = C_{25}H_{30}P^+$; $DTPP^+ = C_{30}H_{40}P^+$; $CTPP^+ = C_{34}H_{48}P^+$; PP = $(C_3H_6)_n$; Stilbene = $C_{14}H_{12}$; Anthracene =$C_{14}H_{10}$.

The fast neutron stopping power is defined by **Supplementary Equation (1)**:

$$\varphi=1-e^{-\mu x} \quad (1)$$

where μ is the elastic scattering cross-section and x is the thickness of the scintillation screen. Calculate the fast neutron stopping power of different samples when x is taken with the same thickness of 1 mm.

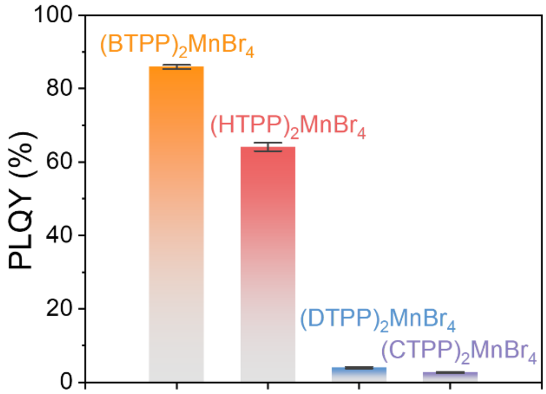

Supplementary Fig. 1. The photoluminescence quantum yield (PLQY) of the single-cation metal halide transparent scintillation screens. Error bars are presented as mean ± standard deviation (SD), n = 3 presents three independent experiments.

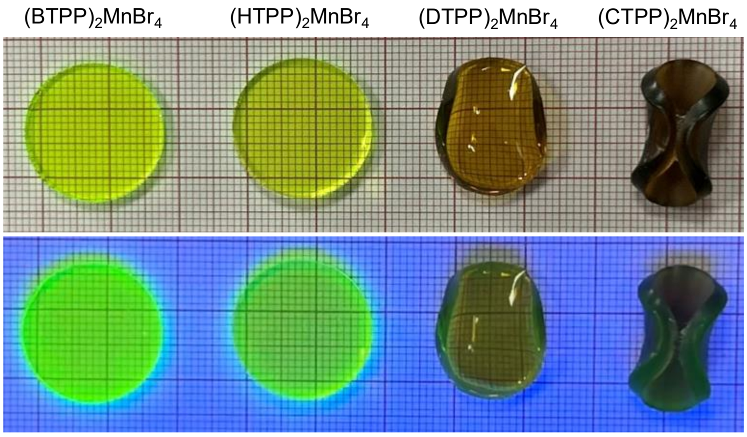

Supplementary Fig. 2. Photographic comparison of the single-cation metal halide transparent scintillation screens with varying mechanical toughness (up: under visible light; down: under 365 nm ultraviolet (UV) light).

Guided by the design criteria for fast neutron scintillation screens, we prioritized selecting hybrid metal halide scintillators with high PLQY. Our investigation revealed that cation size significantly impacts luminescent properties[1, 2]. We systematically tested several single-cation metal halide scintillators, including $(BTPP)_2MnBr_4$, $(HTPP)_2MnBr_4$, $(DTPP)_2MnBr_4$, $(CTPP)_2MnBr_4$ (Supplementary Fig. 1). Among these, $(BTPP)_2MnBr_4$ has the highest PLQY of 85.56%. Moreover, as the branched alkyl chain length increased, we observed a trade-off: mechanical toughness improved, beneficial for scintillation screen durability; however, sample color gradually deepened, increasing self-absorption, and decreasing PLQY (Supplementary Fig. 2). This trade-off highlighted that longer branched alkyl chains enhance mechanical robustness but compromise PLQY (Supplementary Fig. 1-2).

Balancing these competing demands is critical for practical fast neutron scintillation screens, which require both high light output and mechanical integrity. To achieve this balance, we explored combined $BTPP^+$ with other hydrogen-rich cations like $HTPP^+$, $DTPP^+$, $CTPP^+$, which feature long branched alkyl chain. These cations increase hydrogen density, enhancing neutron absorption, while simultaneously improving mechanical properties.

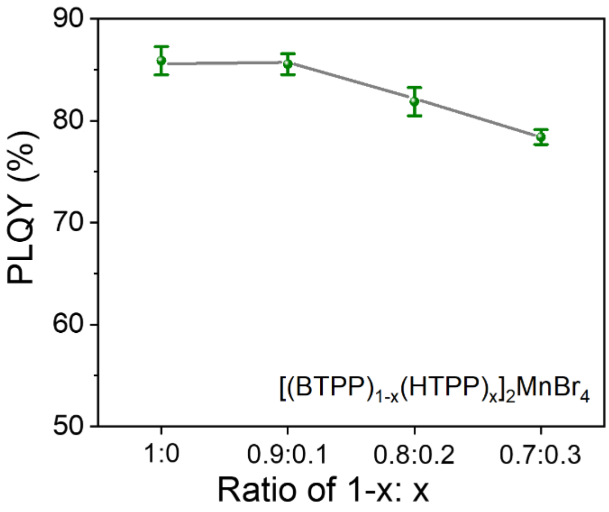

Supplementary Fig. 3. The PLQY of $[(BTPP)_{1-x}(HTPP)_x]_2MnBr_4$ (1-x: x = 1: 0; 0.9: 0.1; 0.8: 0.2; 0.7: 0.3) (Error bars are presented as mean ± SD, n = 3 presents three independent experiments).

We prepared $[(BTPP)_{1-x}(HTPP)_x]_2MnBr_4$ in different ratios: 1-x: x = 1: 0; 0.9: 0.1; 0.8: 0.2; 0.7: 0.3, and measured their PLQY to evaluate their optical performance. It can be seen that the PLQY is highest at 1-x: x = 0.9: 0.1, which is $(BTPP)_{1.8}(HTPP)_{0.2}MnBr_4$. Therefore, we chose 1-x: x = 0.9: 0.1 as the optimal ratio for subsequent experiments and prepared a series of samples with different doped ($(BTPP)_{1.8}(HTPP)_{0.2}MnBr_4$/ $(BTPP)_{1.8}(DTPP)_{0.2}MnBr_4$/ $(BTPP)_{1.8}(CTPP)_{0.2}MnBr_4$), to improve toughness while ensuring fluorescence intensity.

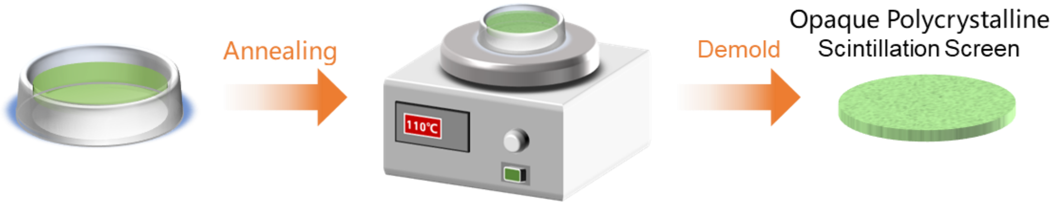

Supplementary Fig. 4. The synthesis process of opaque polycrystalline scintillation screens.

During the experiment, we found that the longer the doped chain, the less possible crystallize, so only $(BTPP)_2MnBr_4$ and $(BTPP)_{1.8}(HTPP)_{0.2}MnBr_4$ polycrystalline scintillation screens were prepared.

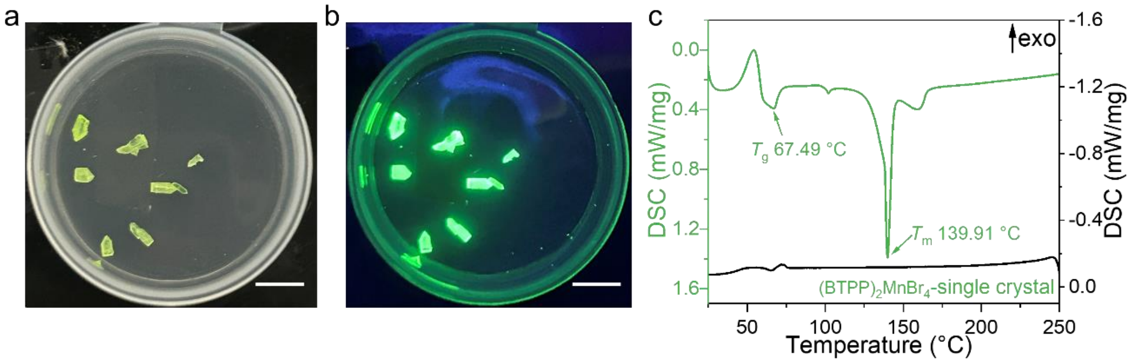

Supplementary Fig. 5. Basic Information of (BTPP)$_2$MnBr$_4$ single crystal. a, The photo under visible light. **b**, The photo under 365 nm UV light. Scale bars: 5 mm. **c,** The differential scanning calorimetry (DSC) of (BTPP)$_2$MnBr$_4$ single crystal.

The millimeter scale (BTPP)$_2$MnBr$_4$ single crystals exhibited green emission under 365 nm UV irradiation. The crystal is in the $P2_1/n$ space group of the monoclinic system with lattice parameters of a = 14.6143 Å, b = 14.8544 Å, and c = 20.6413 Å. The $[MnBr_4]^{2-}$ tetrahedra are spatially isolated and surrounded by large cations $BTPP^+$, forming a periodic 0D structure. The melting range of the single crystal is approximately 137.86-143.57 °C, with a peak melting temperature at 139.91 °C, and the subsequent endothermic event is attributed to post-melting structural evolution.

Supplementary Table 2. Single crystal X-ray diffraction (SCXRD) data of $(BTPP)_2MnBr_4$ single crystal.

Compound	$(BTPP)_2MnBr_4$
Formula	$(C_{22}H_{24}P)_2MnBr_4$
Formula weight	1,013.34
Temperature/K	180
Crystal system	monoclinic
Space group	$P2_1/n$
a/Å	14.6143(3)
b/Å	14.8544(3)
c/Å	20.6413(4)
α/°	91.740(2)
β/°	92.283(2)
γ/°	99.025(2)
Volume/Å^3	4,418.92(16)
Z	4
ρ_{calc}g/cm^3	1.523
μ/mm^{-1}	4.017
F (000)	2,028.0
Radiation	MoKα (λ = 0.71073 Å)
2Θ max for data collection/°	61.438
Reflections collected	23,226
Independent reflections	16,177
Goodness-of-fit on F^2	1.012

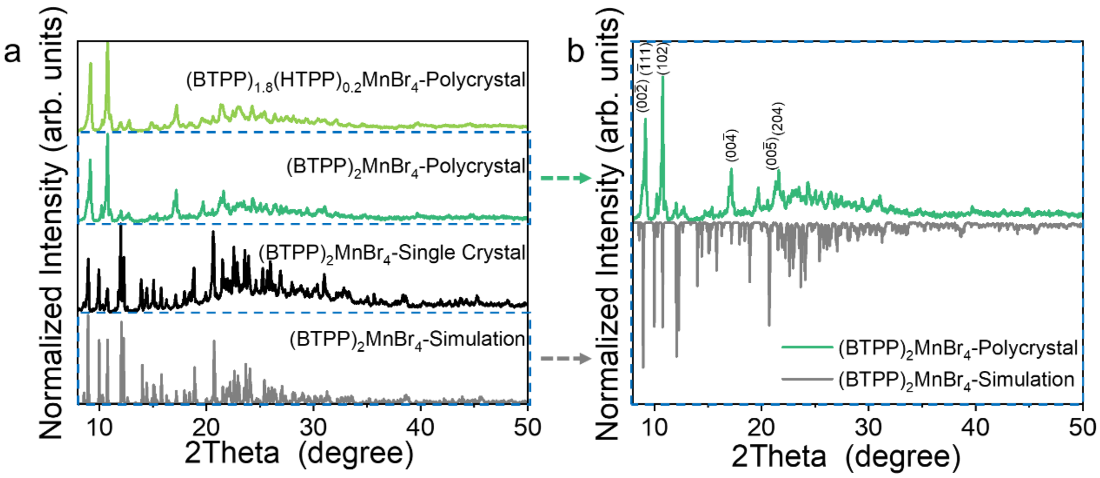

Supplementary Fig. 6. The comparison of powder X-ray diffraction (PXRD) and SCXRD simulation. a, PXRD of $(BTPP)_{1.8}(HTPP)_{0.2}MnBr_4$/ $(BTPP)_2MnBr_4$ polycrystal, the PXRD and SCXRD simulation of $(BTPP)_2MnBr_4$ single crystal. **b**, Enlarged and detailed the comparison between the PXRD of the $(BTPP)_2MnBr_4$ polycrystal and SCXRD simulation of $(BTPP)_2MnBr_4$ single crystal.

The PXRD of $(BTPP)_2MnBr_4$ single crystal exhibits excellent alignment with the peaks simulated from SCXRD. As expected, the polycrystalline samples of $(BTPP)_{1.8}(HTPP)_{0.2}MnBr_4$ and $(BTPP)_2MnBr_4$ display variations in peak intensity due to preferred orientation[3]. Specifically, some diffraction peaks (such as 2θ = 8.57°, 8.95°, 10.8°, 17.2°, 21.5°, 21.6°) are relatively stronger, indicating the preferential crystal growth orientations along the (00$\bar{2}$), ($\bar{1}$11), (102), (00$\bar{4}$), (00$\bar{5}$), (204) planes during the crystallization process[4], which indicates that a small amount of doping long chain $HTPP^+$ has little effect on their crystal structure[5].

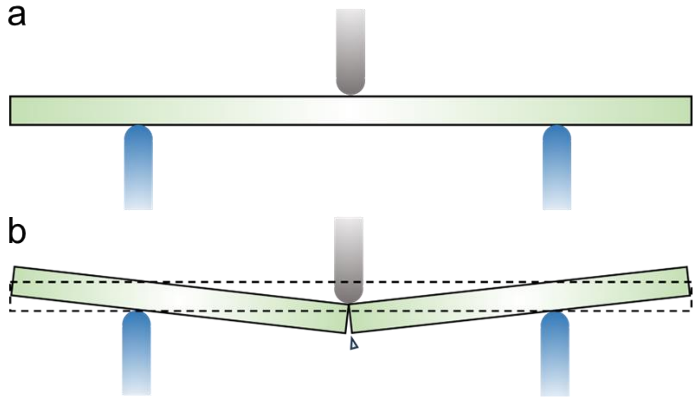

Supplementary Fig. 7. Schematic of three-point bending test. a, Initial shape. **b**, Deflected shape.

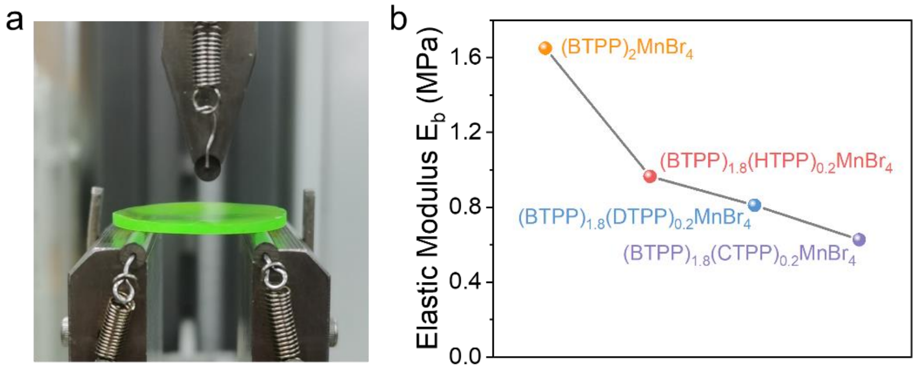

Supplementary Fig. 8. The three-point bending test. a, The photo of test. **b**, The gradually decreasing elastic modulus of scintillation screen showing an increasing mechanical toughness.

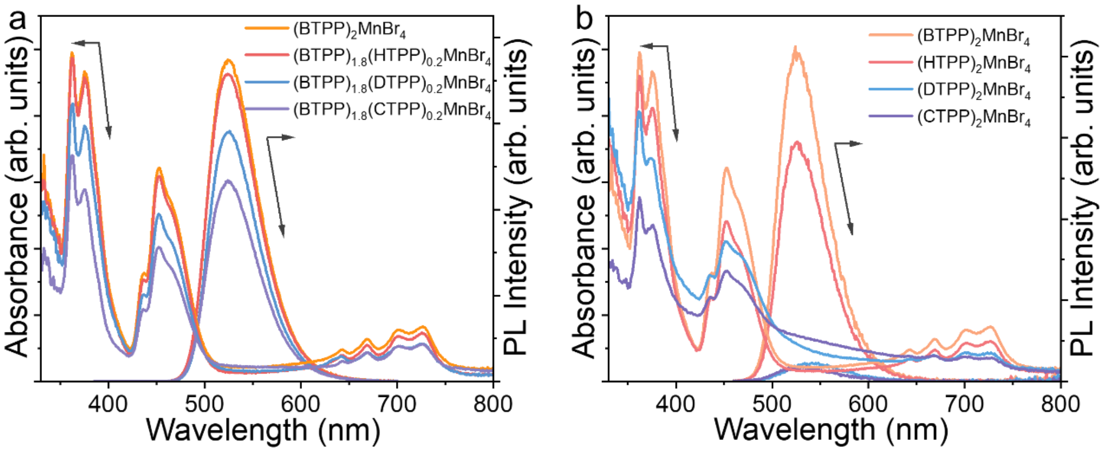

Supplementary Fig. 9. UV absorption and PL emission of different transparent scintillation screen samples. The UV-vis spectra and photoluminescence (PL) spectra of the **a,** $(BTPP)_2MnBr_4$, $(BTPP)_{1.8}(HTPP)_{0.2}MnBr_4$, $(BTPP)_{1.8}(DTPP)_{0.2}MnBr_4$, $(BTPP)_{1.8}(CTPP)_{0.2}MnBr_4$ and **b,** $(BTPP)_2MnBr_4$, $(HTPP)_2MnBr_4$, $(DTPP)_2MnBr_4$, $(CTPP)_2MnBr_4$.

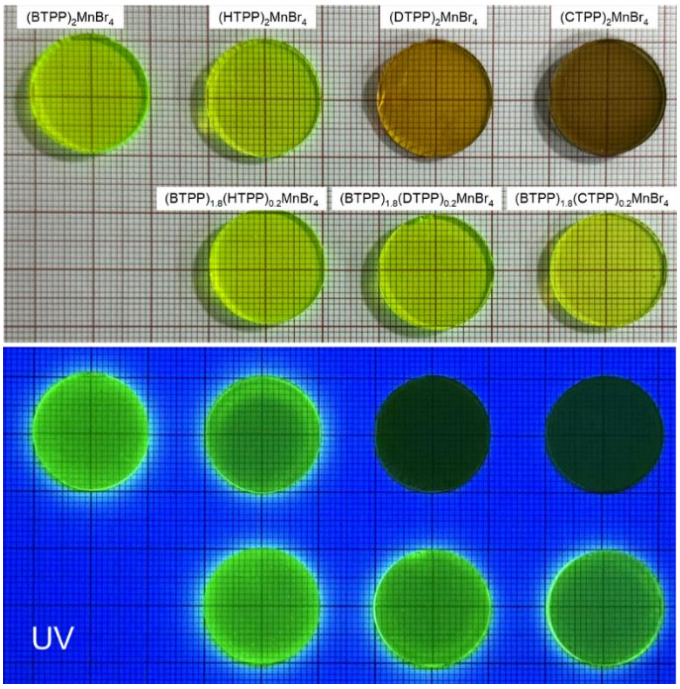

Supplementary Fig. 10. Photos of different transparent scintillation screens under visible and UV light.

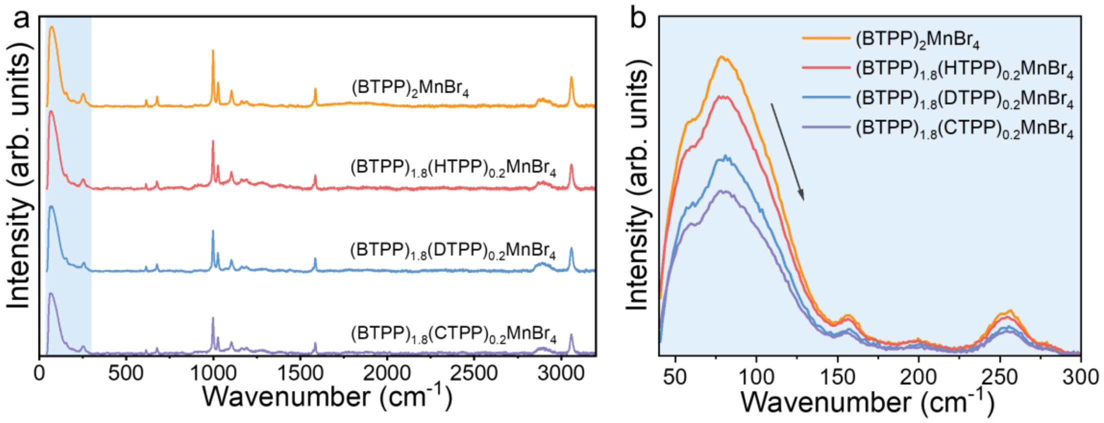

Supplementary Fig. 11. Raman spectra of different transparent scintillation screen samples. **a**, Raman spectra (40-3200 cm^{-1}) and **b**, the enlarged Raman spectra (40-300 cm^{-1}) of the blue-shaded region in (a), showing the vibrational characteristics of Mn-Br in glassy $(BTPP)_2MnBr_4$, $(BTPP)_{1.8}(HTPP)_{0.2}MnBr_4$, $(BTPP)_{1.8}(DTPP)_{0.2}MnBr_4$, $(BTPP)_{1.8}(CTPP)_{0.2}MnBr_4$.

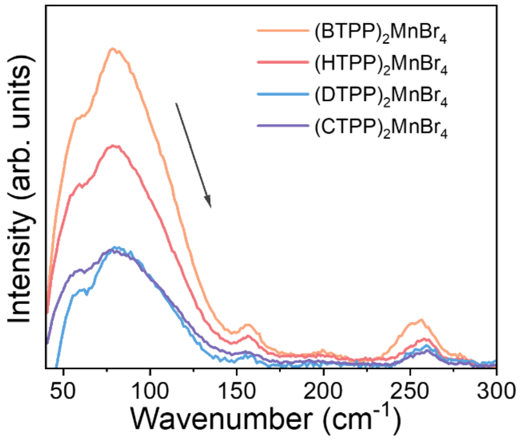

Supplementary Fig. 12. Raman spectra of the single-cation metal halide transparent scintillation screen samples.

As the length of the doped cation branched alkyl chains increases, the absorption intensity of the glass at approximately 365 nm and 450 nm significantly weakens, indicating a decline in the absorption capacity of excitation light, thereby leading to a reduction in excitation efficiency and a subsequent attenuation of emission intensity (Supplementary Fig. 9). On the other hand, in the pure cationic metal halide system (Supplementary Fig. 9b), we discovered that as the length of the cation branched alkyl chains increases, the absorption intensity in the 500-600 nm band gradually enhances, intensifying the self-absorption effect on the green light emission (525 nm) of $[MnBr_4]^{2-}$, further suppressing the output of effective photons. Consequently, the luminescence intensities of $(DTPP)_2MnBr_4$ and $(CTPP)_2MnBr_4$ significantly decrease. The photos (Supplementary Fig. 10) also exhibit the same pattern: as the length of the doped branched alkyl chains increases, the sample color gradually deepens (changing from green to yellow-green under visible light), further indicating the enhancement of the self-absorption effect.

In addition, to further investigate the changes in the luminescent centers, we carried out quantitative Raman spectroscopy tests (Supplementary Fig. 11-12), where the peak intensity is positively correlated with the concentration of the corresponding structure. Within the range of 500-3200 cm^{-1}, we detected the vibration modes of organic cations, and in the low wavenumber range of 40-300 cm^{-1}, the Mn-Br characteristic vibration of the $[MnBr_4]^{2-}$ units was observed[6]. In Supplementary Fig. 11b and Supplementary Fig. 12, as the length of the doped branched alkyl chains increases, the vibration mode of the Mn-Br characteristic vibration peak in the low wavenumber region (40-300 cm^{-1}) becomes non-Gaussian gradually, and weakens in intensity. This evolution clearly demonstrates that long-chain cations cause dilution and excessive spatial separation of $[MnBr_4]^{2-}$ luminescent units, leading to a significant suppression of luminescence efficiency [1, 7, 8].

Thus, with the increase in the length of the doped organic cation chains, the PLQY of $HTPP^+$ doped remains unchanged, while the PLQY of doped long-chain cations $DTPP^+$ and $CTPP^+$ decreases rapidly, which is attributed to the balance among self-absorption effect, steric hindrance, and the density of luminescent centers.

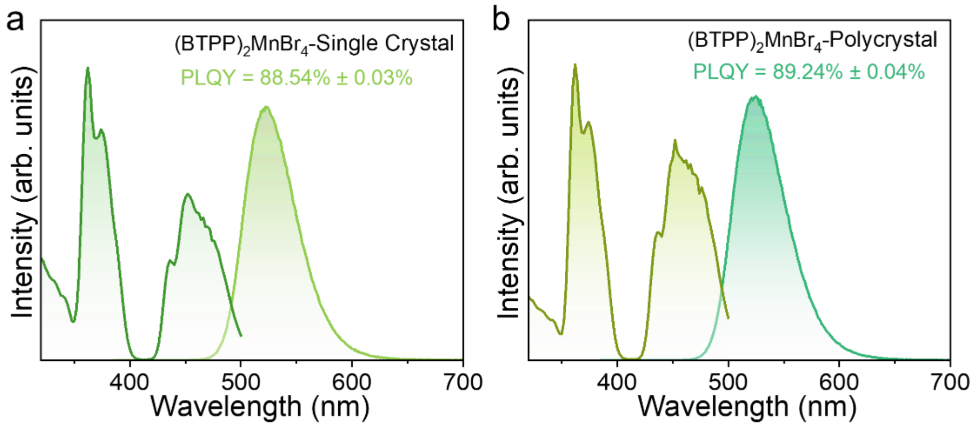

Supplementary Fig. 13. The optical properties of $(BTPP)_2MnBr_4$ crystals. The PL, PL excitation (PLE), and PLQY of **a**, single crystal and **b**, polycrystal.

$(BTPP)_2MnBr_4$ single crystal and polycrystal provide PLQY of 88.54% and 89.24%, respectively, which is due to the optimal Mn-Mn distance in the crystal structure compared to transparent media, avoiding PL quenching caused by Mn-Mn energy transfer, thereby demonstrating higher PLQY[2].

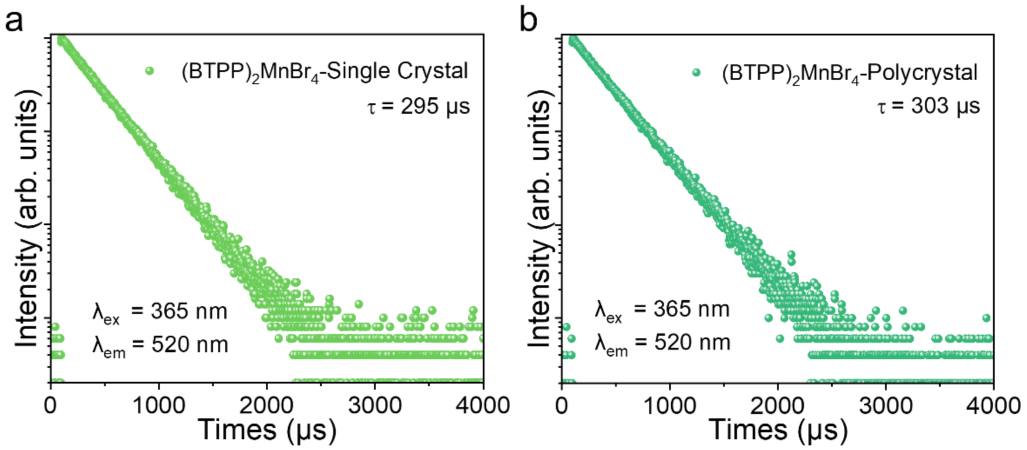

Supplementary Fig. 14. The time-resolved PL (TRPL) of $(BTPP)_2MnBr_4$ crystals. a, Single crystal. **b,** Polycrystal.

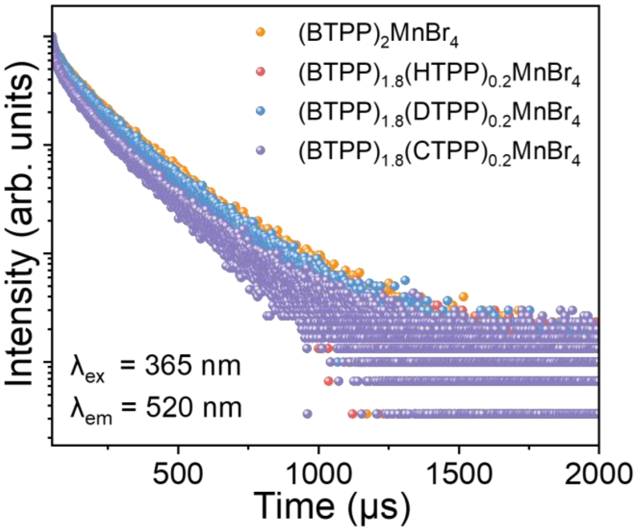

Supplementary Fig. 15. The TRPL of different transparent scintillation screen samples.

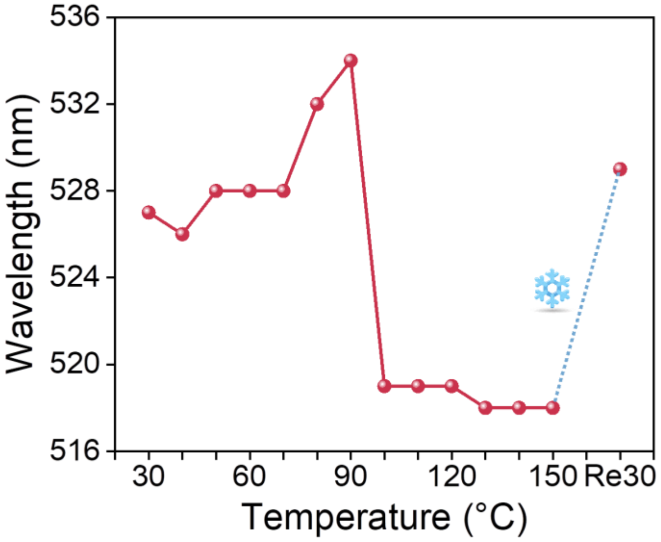

Supplementary Fig. 16. The corresponding PL peak position as a function of temperature (The snowflake represents quenching to RT).

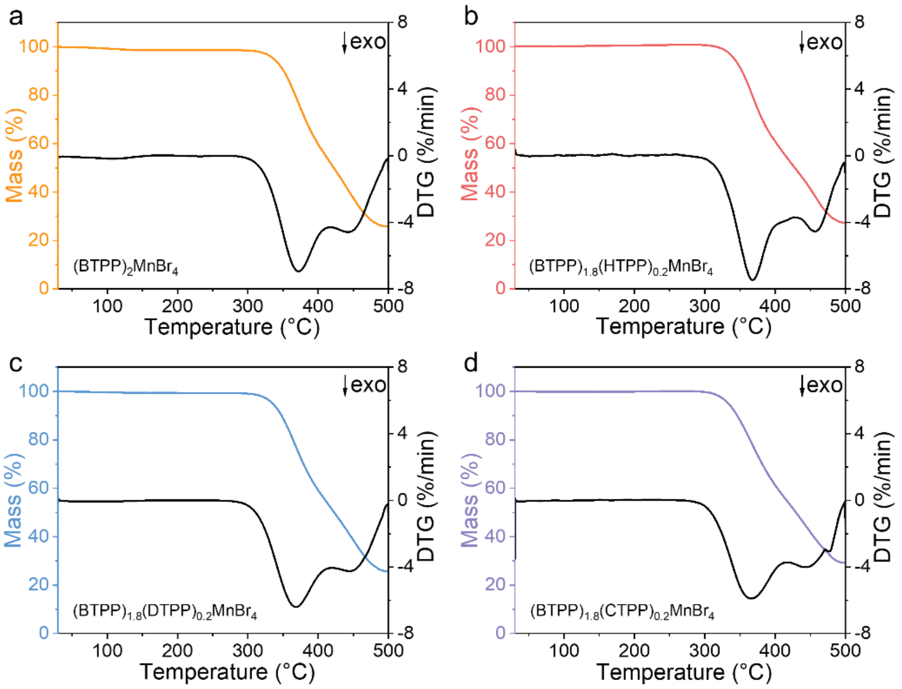

Supplementary Fig. 17. The thermogravimetric (TG) and derivative thermogravimetric (DTG) of different transparent scintillation screen samples. a, $(BTPP)_2MnBr_4$, **b**, $(BTPP)_{1.8}(HTPP)_{0.2}MnBr_4$, **c**, $(BTPP)_{1.8}(DTPP)_{0.2}MnBr_4$, **d**, $(BTPP)_{1.8}(CTPP)_{0.2}MnBr_4$.

Supplementary Fig. 17 shows the result of TG and DTG for four transparent media. The starting decomposition temperature for all of them is 300 °C, which means that the reaction and testing temperatures were kept below 300 °C to prevent decomposition.

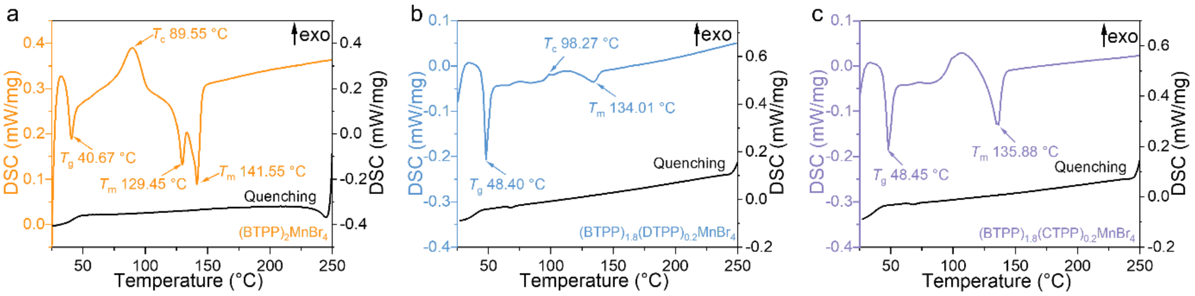

Supplementary Fig. 18. The DSC of different transparent scintillation screen samples. a, $(BTPP)_2MnBr_4$, **b**, $(BTPP)_{1.8}(DTPP)_{0.2}MnBr_4$, **c**, $(BTPP)_{1.8}(CTPP)_{0.2}MnBr_4$.

The T_c of $(BTPP)_2MnBr_4$ is 89.55 °C from DSC result (Supplementary Fig. 18a). Yet the last two show no obvious crystallization peaks, indicating that the longer the branched length of the doped alkyl chain, the weaker the crystallization ability of the medium (Supplementary Fig. 18b-c), which is consistent with the crystallization experiment phenomenon mentioned above.

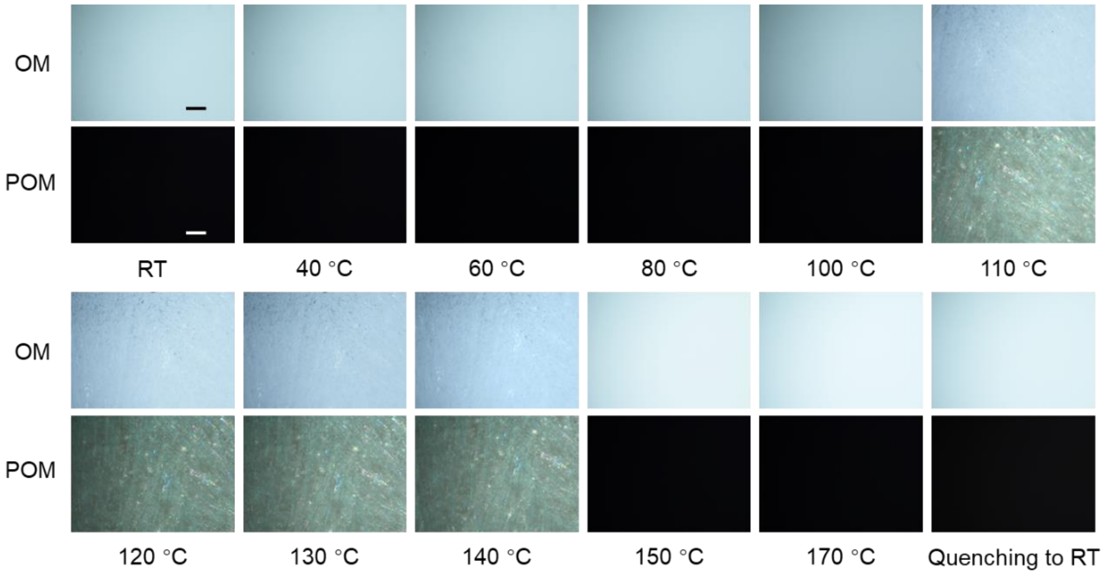

Supplementary Fig. 19. Optical microscopy (OM) and polarized optical microscopy (POM) of $(BTPP)_{1.8}(HTPP)_{0.2}MnBr_4$ during melting and recovery. The POM changed with heating temperature and quenching to room temperature (RT). Scale bars: 100 μm.

The field of view under the POM changes from dark to bright at 110 °C, which is a typical birefringence phenomenon indicating crystallization. At 150 °C, the field of view turned dark, indicating that $(BTPP)_{1.8}(HTPP)_{0.2}MnBr_4$ had completely melted into a liquid. After quenching to RT, the field of view remained dark, implying the $(BTPP)_{1.8}(HTPP)_{0.2}MnBr_4$ solidified to glassy state. The conversion process is consistent with temperature change tests in the main text.

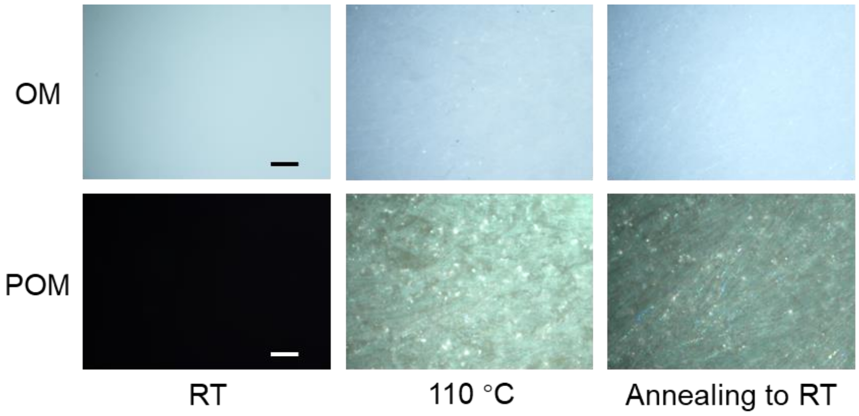

Supplementary Fig. 20. OM and POM of $(BTPP)_{1.8}(HTPP)_{0.2}MnBr_4$ transparent media during the crystallization process. The POM changed with heating up from RT to 110 °C and then annealing down to RT. Scale bars: 100 μm.

After crystallization occurs at the temperature of 110 °C, the opaque polycrystalline sample could be obtained by directly annealing to RT. The conversion process is consistent with temperature change tests (Fig. 3d) in the main text.

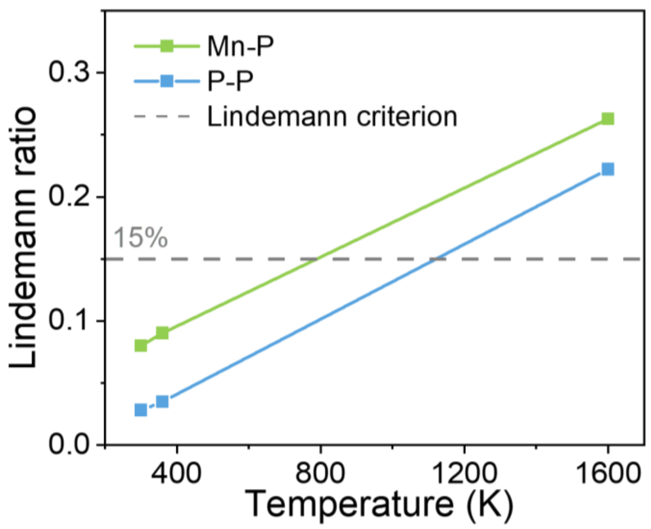

Supplementary Fig. 21. Generalized Lindemann ratio of Mn-P and P-P peak in radial distribution function (RDF).

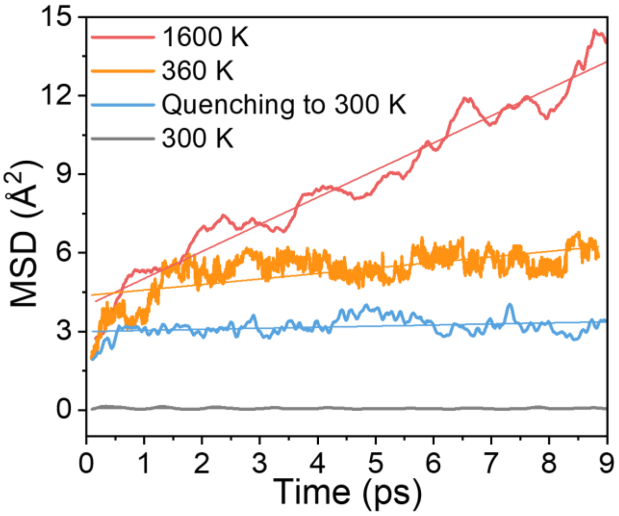

Supplementary Fig. 22. The mean square displacement (MSD) *vs.* simulation time at various temperatures (300 K,1600 K, 360 K, and quenching to 300 K).

Ab initio molecular dynamics (AIMD) simulations were performed to determine the MSD at various temperature, allowing for the computation of diffusion coefficients through Einstein relation. At 1600 K, the diffusion rate of the molecule at 10 ps is 1.03 Å^2 ps^{-1}, the displacement is about 10.3 Å^2 within 10 ps. At 360 K, the diffusion rate is 0.21 Å^2 ps^{-1}. When quenching to 300 K (glassy state), the diffusion rate is 0.04 Å^2 ps^{-1}, and the initial single crystal cell (300 K) is 0 Å^2 ps^{-1}. It indicates that as the temperature increases, the diffusion coefficient of the sample gradually increases, and the sample gradually melts into a liquid.

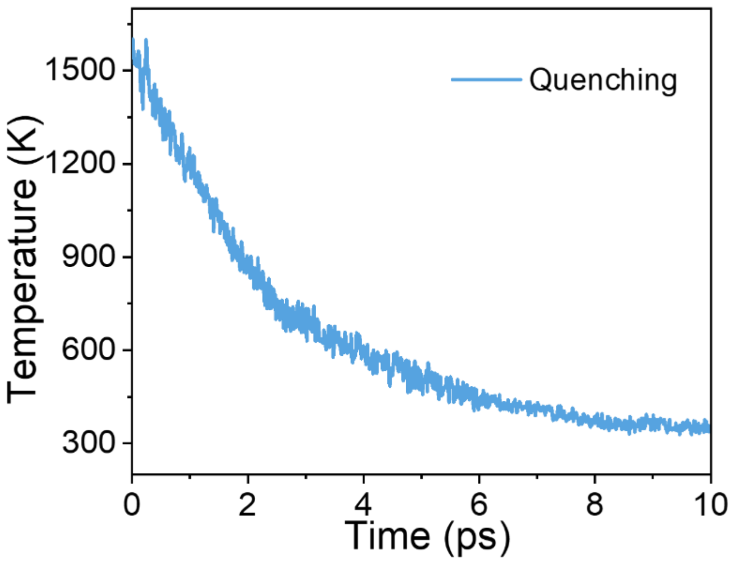

Supplementary Fig. 23. System temperature *vs*. simulation time in the quenching process. The simulation time scale for quenching from molten state (1600 K) to glassy state (300 K) is 10 ps.

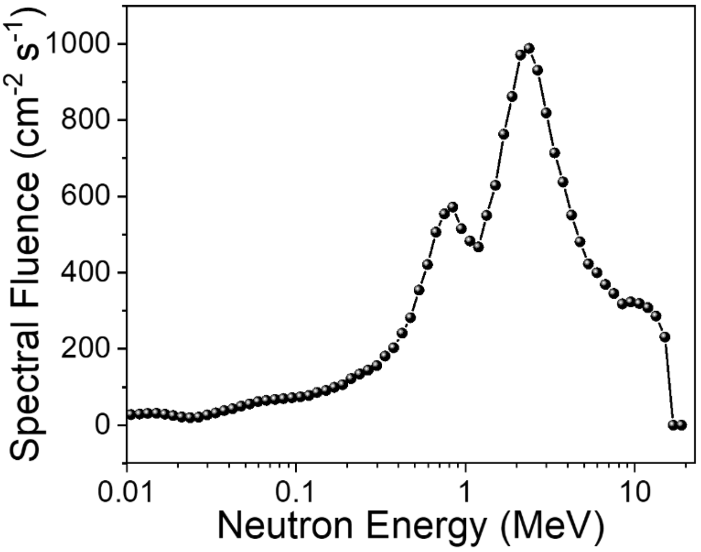

Supplementary Fig. 24. The energy spectra of the fast neutrons in the experiment.

The energy spectra of the fast neutron used in the experiment are extracted from the reactor. The average energy of the fast neutron spectra is 2.69 MeV, produced by thermal neutron induced U^{235} fission. To ensure the purity of the fast neutron beam, a B^{10} filter was employed to effectively remove thermal and epithermal neutrons with energy below 0.01 MeV.

Supplementary Note 1. Calculation method for light absorption length

The absorption length was determined from the attenuation of absorption, following the standard Beer–Lambert[9], while accounting for the influence of reflection on the absorption process, defined by **Supplementary Equation (2)**:

$$\lambda_L = \frac{L/\ln 10}{A+2\log(1-R(\lambda))} \tag{2}$$

where: λ_L: absorption length; L: screen thickness; A: attenuation; $R(\lambda)$: reflectivity, calculated by Fresnel equation.

The attenuation, A, is defined by **Supplementary Equation (3)**:

$$A = -\log\left(\frac{I}{I_0}\right) \tag{3}$$

where: I: the intensity of light passing through the sample; I_0: the intensity of the reference beam.

The transmittance of scintillation screens with varying thicknesses was measured using a Cary 7000 spectrophotometer to calculate the attenuation corresponding to each thickness.

The Fresnel equation calculates reflectivity by **Supplementary Equation (4)**:

$$R(\lambda) = \left(\frac{n_1(\lambda)-n_2(\lambda)}{n_1(\lambda)+n_1(\lambda)}\right)^2 \tag{4}$$

where: $n_1(\lambda)$ is the refractive index of air, the refractive index of air is approximately 1. $n_2(\lambda)$ is the refractive index of the screen. $n_2(\lambda)$ is 1.660 for $(BTPP)_{1.8}(HTPP)_{0.2}MnBr_4$ and 1.548 for ZnS (Ag): PP).

Therefore, we obtained a linear formula related to the length of light absorption defined by **Supplementary Equation (5)**:

$$L = \ln 10\,\lambda_L A + 2\lambda_L \ln(1-R(\lambda)) \tag{5}$$

And linear fitting using attenuation A and sample thickness as the x- and y- coordinates were performed, respectively. By calculating the slope of the linear formula, the fitted light absorption length value λ_L can be obtained.

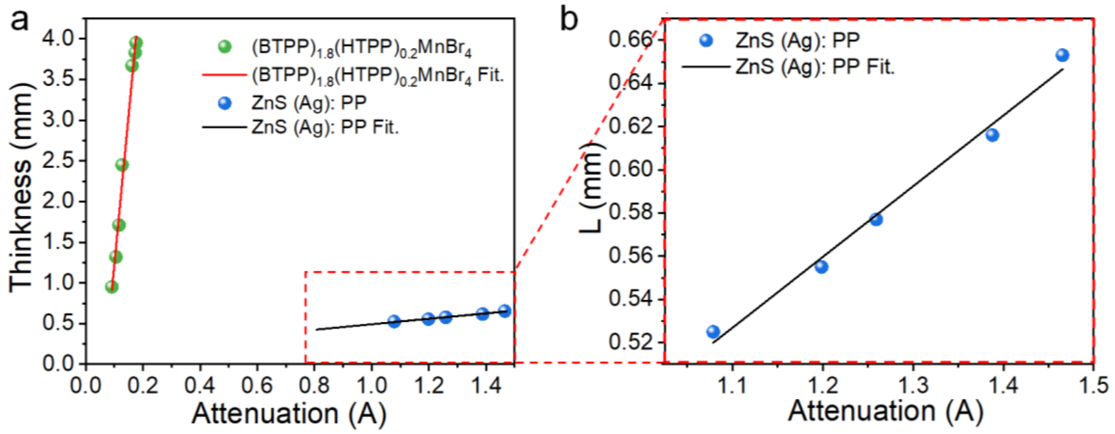

Supplementary Fig. 25. Linear fitting diagrams for attenuation and sample thickness. **a,** $(BTPP)_{1.8}(HTPP)_{0.2}MnBr_4$ and ZnS (Ag): PP. **b,** Magnified view of ZnS (Ag): PP.

Based on Supplementary Note 1 and Supplementary Fig. 25, the slope of the linear formula is 36.85 for $(BTPP)_{1.8}(HTPP)_{0.2}MnBr_4$ and 0.3274 for ZnS (Ag): PP. The light absorption length is approximately: $\lambda_L \approx 15.9$ mm for $(BTPP)_{1.8}(HTPP)_{0.2}MnBr_4$ and $\lambda_L \approx 0.142$ mm for ZnS (Ag): PP.

Supplementary Note 2. Method for calculating the energy deposition efficiency of secondary particles in neutron-induced processes

The neutron energy deposition efficiency is defined by **Supplementary Equation (6)**,

$$\delta=\frac{E_n}{E_0} \tag{6}$$

where E_0 is the total energy of fast neutron incidence and E_n is the neutron deposition energy.

The recoil proton energy deposition efficiency is defined by **Supplementary Equation (7)**,

$$\sigma=\frac{E_{r2}}{E_n} \tag{7}$$

where E_n is the neutron deposition energy and E_{r2} is the recoil proton deposition energy.

The secondary γ-ray energy deposition efficiency is defined by **Supplementary Equation (8)**:

$$\rho=\frac{E_{g2}}{E_n} \tag{8}$$

where E_n is the neutron deposition energy and E_{g2} is the secondary γ-ray deposition energy.

Supplementary Table 3. Summary of the physical process of the interaction between fast neutrons and $(BTPP)_{1.8}(HTPP)_{0.2}MnBr_4$, ZnS (Ag): PP.

Parameters	$(BTPP)_{1.8}(HTPP)_{0.2}MnBr_4$	ZnS (Ag): PP
Total energy of fast neutron incidence (E_0, MeV)	2.69×10^7	2.69×10^7
Neutron deposition energy (E_n, MeV)	3.003×10^5	5.748×10^5
Neutron energy deposition efficiency (δ)	1.116%	2.137%
Generated recoil proton energy (E_{r1}, MeV)	2.688×10^5	5.368×10^5
Recoil proton deposition energy (E_{r2}, MeV)	2.323×10^5	4.831×10^5
Recoil proton energy deposition efficiency (σ)	77.35%	84.05%
Generated secondary γ-ray energy (E_{g1}, MeV)	1.279×10^5	1.688×10^5
Secondary γ-ray deposition energy (E_{g2}, MeV)	229.4	297.0
Secondary γ-ray energy deposition efficiency (ρ)	0.07640%	0.05167%

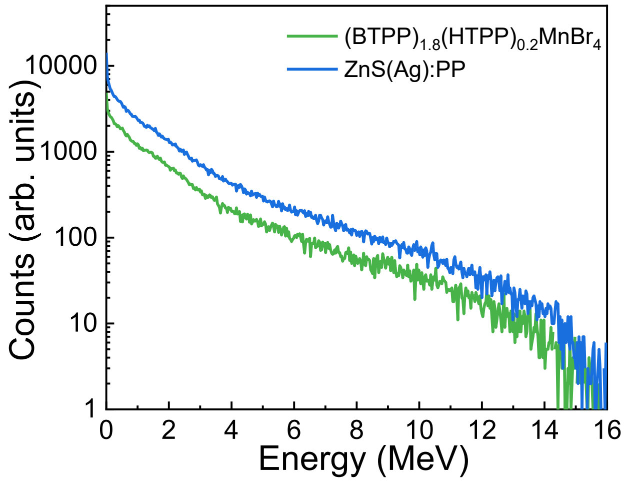

Supplementary Fig. 26. Recoil proton energy spectrum of $(BTPP)_{1.8}(HTPP)_{0.2}MnBr_4$ and ZnS (Ag): PP.

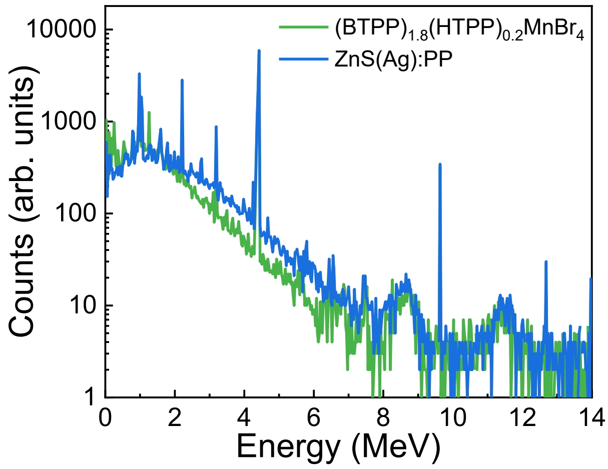

Supplementary Fig. 27. Secondary γ-ray spectrum of $(BTPP)_{1.8}(HTPP)_{0.2}MnBr_4$ and ZnS (Ag): PP.

Supplementary Discussion 1. Energy deposition efficiency of secondary particles during neutron interaction process

During the calculation process, the total energy of fast neutron incidence was 2.69×10^7 MeV. We obtained the neutron deposition energy of ZnS (Ag): PP (5.748×10^5 MeV) is approximately twice that of $(BTPP)_{1.8}(HTPP)_{0.2}MnBr_4$ (3.003×10^5 MeV). This is because hydrogen density affects the elastic scattering cross-section, which in turn affects the deposition of fast neutrons in the scintillation screen. According to Supplementary Equation (6) in Supplementary Note 2, the neutron energy deposition efficiency of $(BTPP)_{1.8}(HTPP)_{0.2}MnBr_4$ (1.116%) is lower than that of ZnS (Ag): PP (2.137%), primarily due to difference in hydrogen density.

Meanwhile, the detailed information on secondary particles generated by interactions between fast neutrons and scintillators was also obtained by simulation. The results confirm that the energy deposited by fast neutrons in the scintillation screens is primarily attributed to recoil protons. The recoil proton energy spectra of different scintillation screens are shown in Supplementary Fig. 26. Additionally, simulations revealed that, apart from recoil protons, secondary γ-rays are produced through neutron inelastic scattering and neutron capture, without the generation of secondary electrons, as illustrated in the energy spectrum (Supplementary Fig. 27). Notably, no β-rays were observed during the simulation of the reaction channel process.

Furthermore, the calculated energy deposition efficiency of recoil protons was found to be 77.35% for $(BTPP)_{1.8}(HTPP)_{0.2}MnBr_4$ and 84.05% for ZnS (Ag): PP, respectively (Supplementary Equation (3)). Then, according to Supplementary Equation (8) in Supplementary Note 2, the energy deposition efficiency of secondary γ-rays was calculated to be 0.07640% for $(BTPP)_{1.8}(HTPP)_{0.2}MnBr_4$ and 0.05167% for ZnS (Ag): PP, respectively. It is important to note that, due to limitations in the current theoretical model, both $(BTPP)_{1.8}(HTPP)_{0.2}MnBr_4$ and ZnS (Ag): PP scintillators were modeled as homogeneous single-component structures, representing idealized values. This modeling approach does not account for potential advantages of single-component systems compared to physical mixtures.

These findings indicate that the recoil proton channel is the primary contributor to the scintillation process. In contrast, the energy deposited by secondary γ-rays is significantly lower and has a relatively minor impact on the overall scintillation process.

Supplementary Table 4. The conversion probability of recoil protons and the grayscale value related data of $(BTPP)_{1.8}(HTPP)_{0.2}MnBr_4$ and ZnS (Ag): PP.

Parameters	$(BTPP)_{1.8}(HTPP)_{0.2}MnBr_4$	ZnS (Ag): PP
**Number of incident fast neutrons (N_0)**	1×10^7	1×10^7
**Number of neutrons that generate recoil protons (N)**	1.278×10^5	2.505×10^5
**The conversion probability of recoil protons (ε)**	1.278%	2.505%
**Number of neutrons reaching the sample*[1]**	10,000	10,000
**Number of neutrons that generate recoil protons*[2]**	127.8	250.5
**Measured grayscale value**	208±5	70±3
**PLQY**	85.54%	31.38%

*[1] Under the detection condition of a neutron flux of 10^7 n cm^{-2} s^{-1}, an imaging size of 50 μm pix^{-1} and an exposure time of 40 s, the number of neutrons reaching the sample was calculated to be 10,000 n pix^{-1}: 10^7 n cm^{-2} s^{-1} × $(50\times10^{-4}$ cm $pix^{-1})^2$ × 40 s = 10,000 n pix^{-1}.

*[2] Number of neutrons reaching the sample is 10,000 n pix^{-1}, The conversion probability of recoil protons (ε) of $(BTPP)_{1.8}(HTPP)_{0.2}MnBr_4$ and ZnS (Ag): PP is 1.278% and 2.505%, respectively. The number of neutrons that generate recoil protons was calculated to be 127.8 and 250.5, respectively: 10,000 × 1.278% = 127.8; 10,000 × 2.505% = 250.5.

The conversion probability of recoil protons is defined by **Supplementary Equation (9)**,

$$\varepsilon=\frac{N_0}{N} \quad (9)$$

where: N_0 is the number of incident fast neutrons and N is the number of neutrons that generate recoil protons (there are cases where one neutron generates multiple recoil protons).

We extracted and calculated the grayscale values from the detection results (Supplementary Table 4). Under the experimental condition of a neutron flux of 10^7 n cm^{-2} s^{-1}, an imaging size of 50 μm pix^{-1} and an exposure time of 40 s, the number of neutrons reaching the sample was calculated as 10,000 n pix^{-1}. Using theoretical calculations of the number of neutrons generating recoil protons (Supplementary Table 4), the conversion probabilities of recoil protons were calculated using Supplementary Equation (9) to be 1.278% for $(BTPP)_{1.8}(HTPP)_{0.2}MnBr_4$ and 2.505% for ZnS (Ag): PP, respectively. Therefore, under the same conditions, the number of neutrons that generate recoil protons is 127.8 n pix^{-1} in $(BTPP)_{1.8}(HTPP)_{0.2}MnBr_4$ and 250.5 n pix^{-1} in ZnS (Ag): PP. Despite the lower neutron interaction and recoil proton generation probabilities for $(BTPP)_{1.8}(HTPP)_{0.2}MnBr_4$, its detected grayscale value is approximately 208, which nearly three times that of ZnS (Ag): PP (grayscale value: 70). This indicates that the light output from $(BTPP)_{1.8}(HTPP)_{0.2}MnBr_4$ is determined by its superior photon statistics, stemming from its higher transparency and efficient photon transmission.

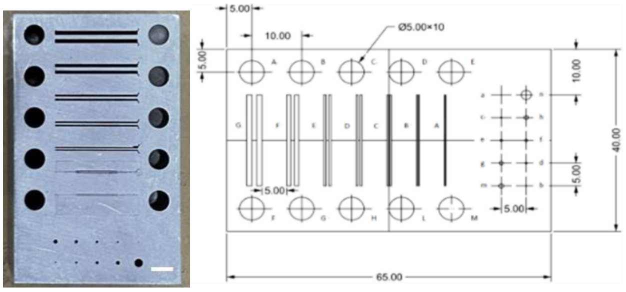

Contrast Sensitivity Test hole depth / mm		Spatial Resolution Test line pair width / mm		Spatial Resolution Test hole diameter / mm	
A	3	A	0.1	a	0.2
B	6	B	0.2	b	0.3
C	9	C	0.3	c	0.4
D	12	D	0.4	d	0.5
E	15	E	0.5	e	0.6
F	18	F	0.8	f	0.7
G	22	G	1	g	0.8
H	25			h	0.9
L	28			m	1
M	30			n	2

Supplementary Fig. 28. Photo and size parameters of standard steel slit plate for resolution testing. Scale bars: 5 mm.

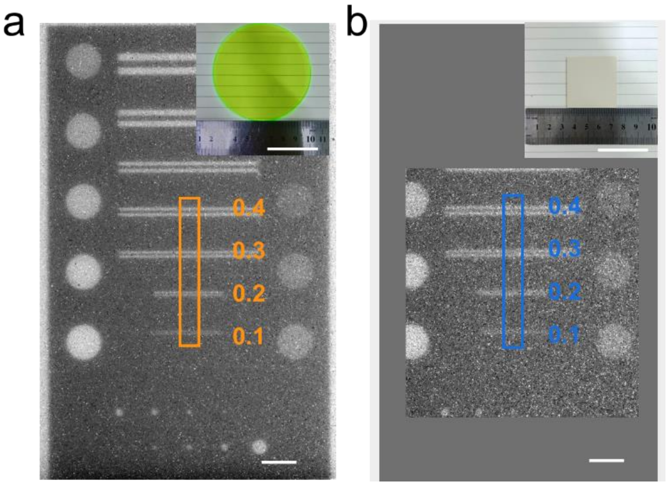

Supplementary Fig. 29. Resolution test with standard sample. a, Using $(BTPP)_2MnBr_4$ transparent scintillation screen. **b,** Using ZnS (Ag): PP commercial screen. Scale bars: 5 mm. The insets show the photos of corresponding scintillation screen. Scale bars: 4 cm. The imaging field of the ZnS (Ag): PP commercial screen is different from others because of its size limitation.

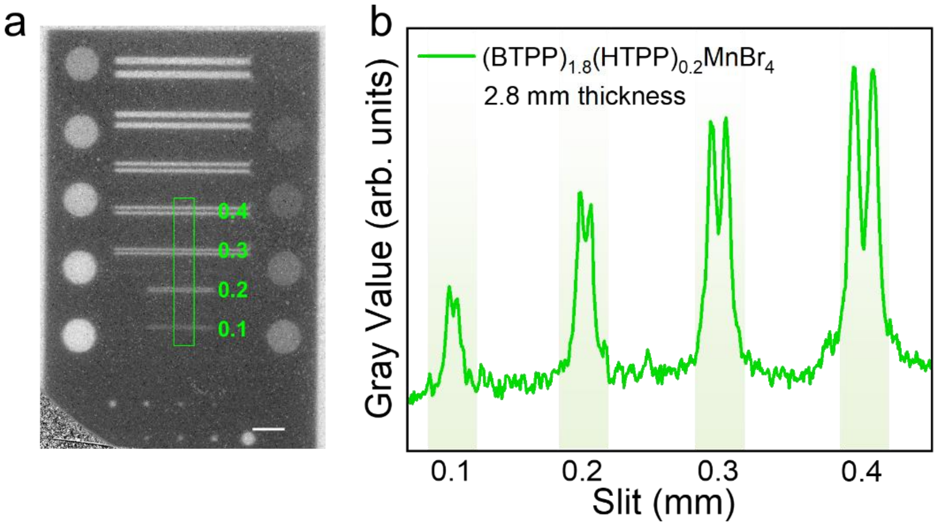

Supplementary Fig. 30. Imaging experiment of 2.8 mm scintillator screen. a, Fast neutron imaging of resolution test standard samples using $(BTPP)_{1.8}(HTPP)_{0.2}MnBr_4$ transparent scintillation screen with a thickness of 2.8 mm and **b,** the extracted relative gray value. Scale bar: 5 mm.

Supplementary Note 3. Calculation of intrinsic resolution in fast neutron imaging using the MTF

In fast neutron imaging, spatial resolution is typically evaluated using resolution templates and gray-scale extraction, rather than directly employing the modulation transfer function (MTF). This is because the non-parallel nature of fast neutron beams requires MTF test templates to have sharp edges and be as thin as possible. However, due to the strong penetration ability of fast neutrons, thin materials cannot fully block neutrons, resulting in reduced image contrast. To establish a quantitative relationship between MTF and spatial resolution, the contrast must be enhanced by increasing the thickness of the mask. Unfortunately, this introduces geometric unsharpness into the measurements, as described by **Supplementary Equation (10)**[10]:

$$G=\frac{d}{L/D} \tag{10}$$

where L is the distance from the neutron source to the image detector; D is the diameter at the exit of the neutron source, and d is the distance from the sample center to scintillation screen.

Therefore, the intrinsic resolution of the scintillation screen can thus be expressed as **Supplementary Equation (11)**:

$$Resolution_{s}\approx\sqrt{Resolution_{\mathrm{MTF}}^{2}\text{-}Resolution_{\mathrm{G}}^{2}} \tag{11}$$

where $Resolution_{s}$ is the intrinsic resolution of the scintillation screen, $Resolution_{\mathrm{MTF}}$ is the MTF value, and $Resolution_{\mathrm{G}}$ refers to the resolution of the geometric unsharpness.

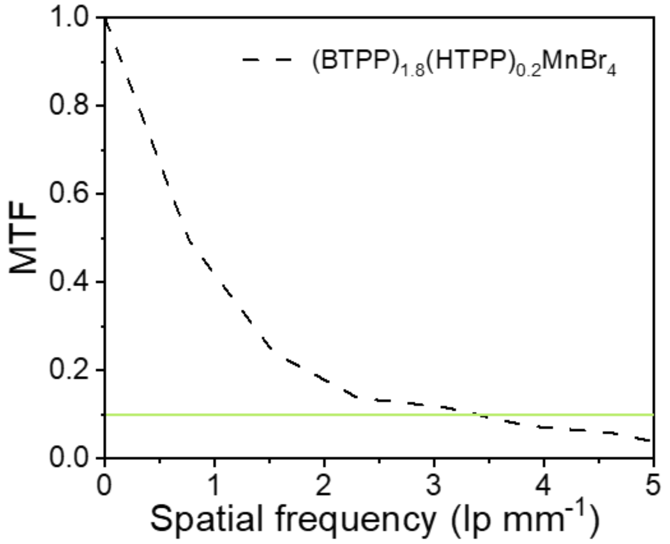

Supplementary Fig. 31. MTF of $(BTPP)_{1.8}(HTPP)_{0.2}MnBr_4$ transparent scintillation screen.

In the experiment, a 3 cm-thick stainless steel block was used as a mask to perform MTF testing on $(BTPP)_{1.8}(HTPP)_{0.2}MnBr_4$ transparent scintillation screen. The results that the spatial resolution is approximately 3.5 lp mm^{-1} at MTF=0.1, corresponding to a slit width of 0.145 mm (Supplementary Fig. 31). Additionally, the L/D ratio of the neutron imaging system used in this

work is 160. The geometric unsharpness G is calculated to be 0.094 mm when d=1.5 cm. Combining these results and Supplementary Note 3, the $Resolution_s$ of the scintillation screen is determined to be 4.55 lp mm^{-1} (approximately 0.11 mm), consistent with the resolution obtained using the resolution template.

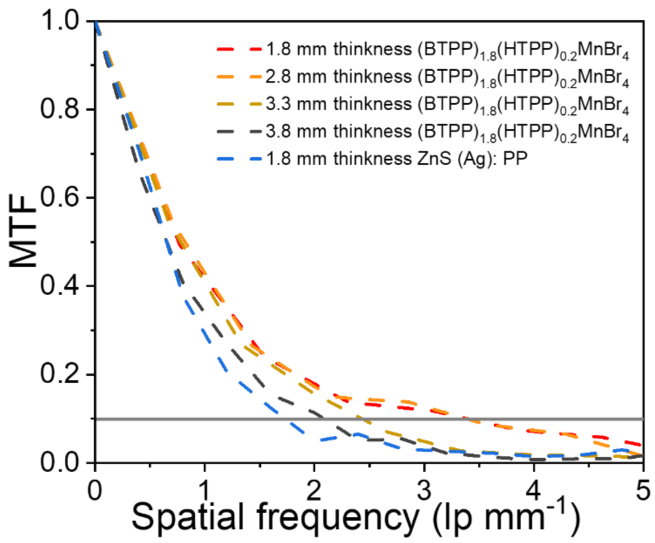

Supplementary Fig. 32. MTF of $(BTPP)_{1.8}(HTPP)_{0.2}MnBr_4$ transparent scintillation screen at various thicknesses, using ZnS (Ag): PP as a reference.

To quantify the relationship between scintillation screen thickness and resolution, MTF measurements for $(BTPP)_{1.8}(HTPP)_{0.2}MnBr_4$ transparent scintillation screen at various thicknesses, using ZnS (Ag): PP as a reference were also conducted (Supplementary Fig. 32). The results show that: When the MTF is increased from 1.8 mm to 2.8 mm, the MTF is almost unchanged, both are 3.5 lp mm^{-1}, combined with geometric unsharpness, the corresponding spatial resolution is about 4.55 lp mm^{-1}. Then the resolution decreases with the increase of the thickness (from 2.8 mm to 3.8 mm). At a thickness of 3.8 mm, the MTF value dropped to 2.1 lp mm^{-1}, corresponding to a spatial resolution of 2.8 lp mm^{-1}, but is still higher than that of ZnS (Ag): PP (1.72 lp mm^{-1} at MTF=0.1, the corresponding spatial resolution is about 1.82 lp mm^{-1}).

Supplementary Table 5. Performance comparison of glassy $(BTPP)_{1.8}(HTPP)_{0.2}MnBr_4$ and reported fast neutron scintillation screens.

Scintillation screen	Type	Screen component	Hydrogen density (n cm^{-3})	PLQY (%)	Thickness (mm)	Neutron light output* (photons/neutron)	Spatial resolution (lp mm^{-1})
**WFC (Waveshift Fiber Converter)[11]**	Opaque screen	two-component	-	-	10	-	0.5
**PZC (Polyethylene-ZnS Converter)[11]**	Opaque screen	two-component	-	-	3	-	> 0.5
**B14E MCPs (Microchannel Plates)[12]**	Opaque screen	two-component	-	-	-	-	2.5
**ZnS (Ag): PP (RC TRITEC, Swiss)[13]**	Opaque screen	two-component	-	-	1.5	-	2
**Stilbene[14]**	Organic	single-component	4.05×10^{22}	65.00	-	~ 26.51% of the ZnS: Cu (PP)	-
**Anthracene[14]**	Organic	single-component	4.32×10^{22}	64.00	-	~ 37.88% of the ZnS: Cu (PP)	-
**BCF-12 (Crytur)[15]**	Plastic fiber screen	two-component	-	-	50	-	0.5-0.7
**PVT (2% X-Flrpic) (Polyvinyl Toluene)[16]**	Plastic	two-component	-	-	3	-	2.28
**PVT (2% Ir-complex)[17]**	Plastic	two-component	-	-	10	-	2.56
**TPE-4Br@PVT[18]**	@PVT plastic	two-component	5.29×10^{22}	53.3	3	-	2.03
**$CsPbBrCl_2$: Mn in hexane [19]**	Nanocrystal solution	two-component	-	53	10	11.24% of the ZnS: Cu (PP)	0.185
**$FAPbBr_3$ in toluene [20]**	Nanocrystal solution	two-component	-	96.2	10	19.3% of the ZnS: Cu (PP)	0.1
**$(BA)_2PbBr_4$ [21]**	@PDMS film	two-component	4.82×10^{22}	-	2.5	86% of the ZnS (Ag)/^{6}LiF	1
**Mn-STA_2PbBr_4 [22]**	Self-standing plate	single-component	9.51×10^{22}	58.58	1.135	79.05% of the ZnS (Ag): PP	0.5
**$(PEA)_2PbBr_4$ [23]**	Single crystal	single-component	1.77×10^{21}	-	1.9	-	2
**Li-$(PEA)_2PbBr_4$ [24]**	Single crystal	single-component	-	-	1	-	1
**$LiInSe_2$ [25]**	Crystal	single-component	-	-	0.528	-	0.32
**$(BTPP)_{1.8}(HTPP)_{0.2}MnBr_4$**	Transparent glass state	single-component	4.18×10^{22}	85.54	1.8	~ 306% of the ZnS (Ag): PP	5
					2.8	~ 531% of the ZnS (Ag): PP	5

*: The fast neutron light output of ZnS (Ag): PP is about 78.90%-84.21% of ZnS (Cu): PP [13].

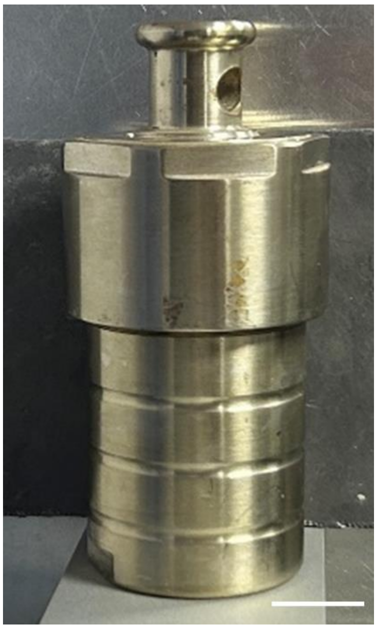

Supplementary Fig. 33. The photo of hydrothermal reactor. Scale bar: 2 cm.

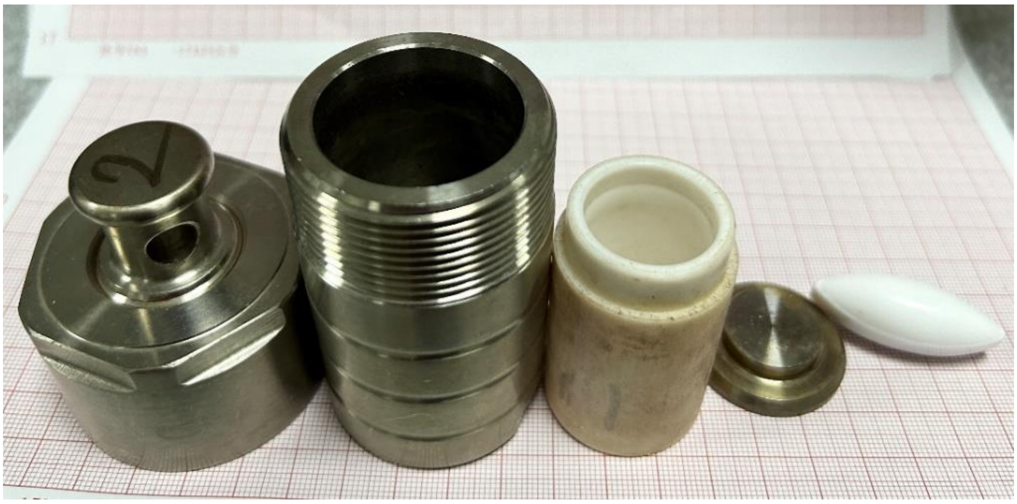

Supplementary Fig. 34. The photo of hydrothermal reactor internal details.

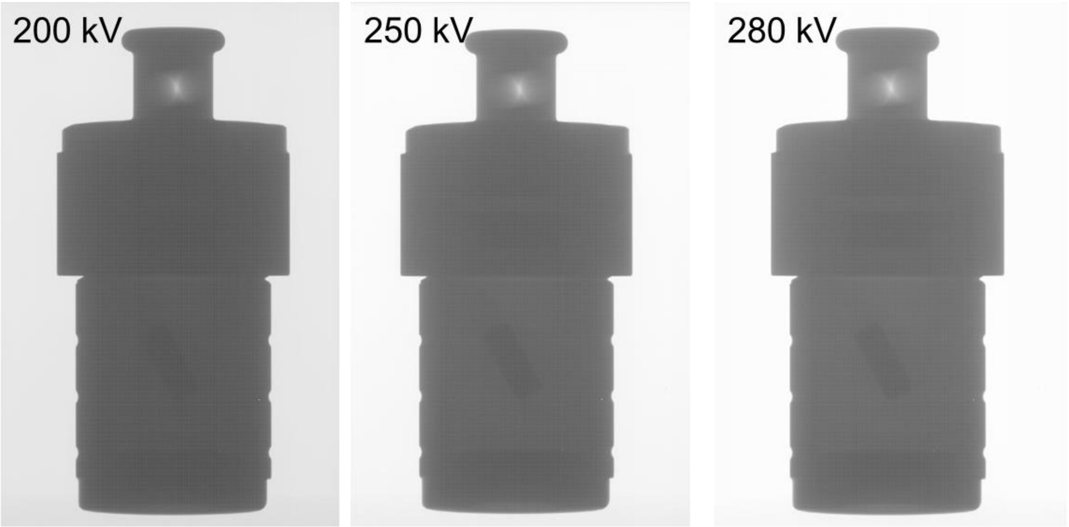

Supplementary Fig. 35. High-energy X-ray industrial computerized tomography (CT) imaging of a complicated scenario. A thick stainless steel hydrothermal reactor with polymer lining containing liquid and a stir bar (200 kV, 250 kV, 280 kV).

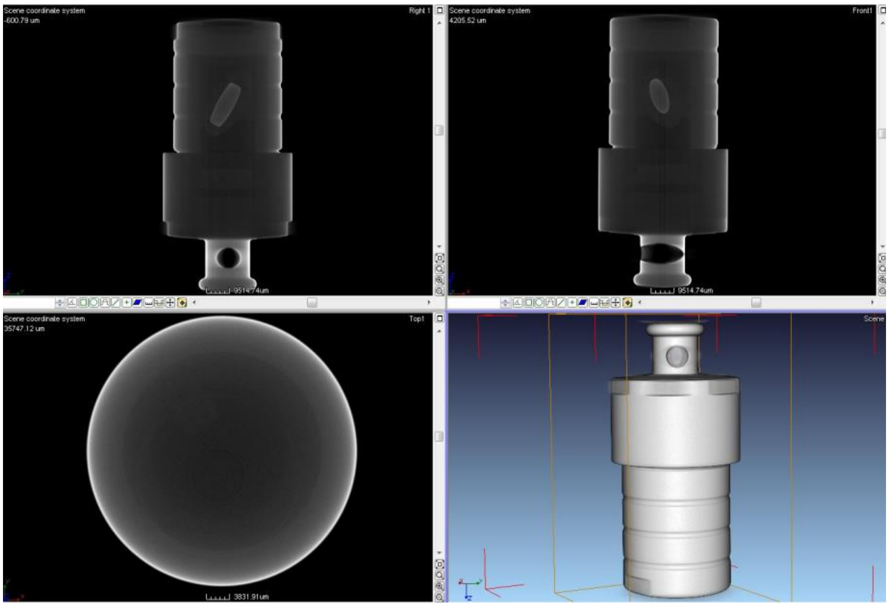

Supplementary Fig. 36. Three-view projections and three-dimensional (3D) reconstructed image of a hydrothermal reactor. High-energy industrial X-ray CT imaging and 3D reconstruction of a thick-walled stainless steel hydrothermal reactor with polymer lining containing liquid and a magnetic stir bar.

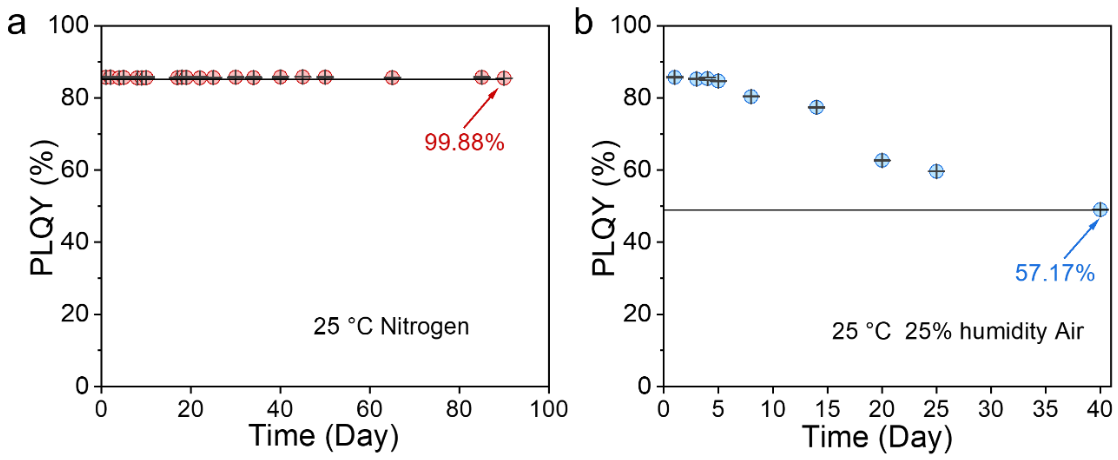

Supplementary Fig. 37. The stability of the PLQY of $(BTPP)_{1.8}(HTPP)_{0.2}MnBr_4$ scintillation screen. a, Under storage conditions (25 °C nitrogen). **b,** Under fast neutron imaging conditions (25 °C and 25% humidity air). (Error bars are presented as mean ± SD, n = 3 presents three independent experiments)

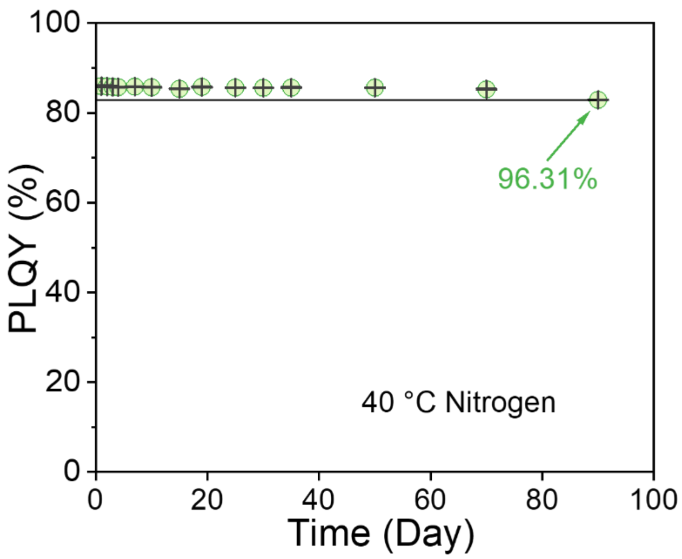

Supplementary Fig. 38. The stability of the PLQY of $(BTPP)_{1.8}(HTPP)_{0.2}MnBr_4$ stored in nitrogen at 40 °C (mean ± SD, n = 3).

Due to the limited operating temperature of the FNR (23-28 °C), we evaluated the effect of elevated temperature on storage stability. When stored in nitrogen at 40 °C, the PLQY of $(BTPP)_{1.8}(HTPP)_{0.2}MnBr_4$ decreased to 96.31% of the initial value after 90 days, indicating minimal impact of higher temperatures during storage.

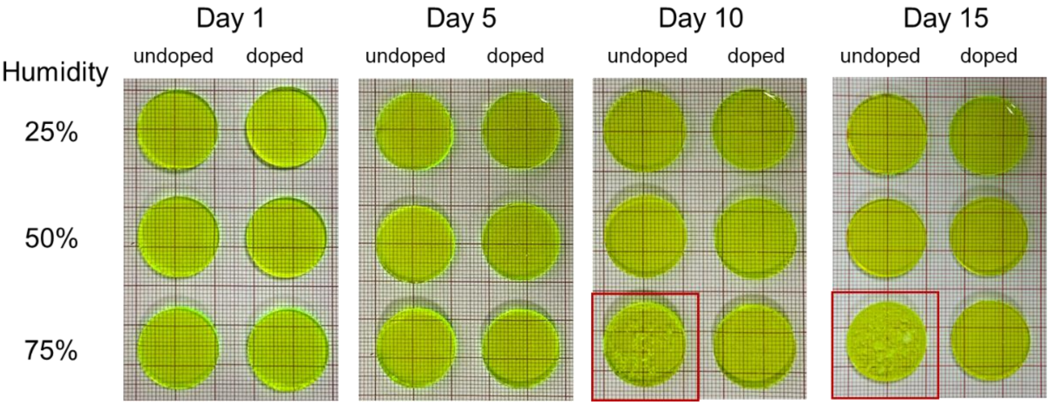

Supplementary Fig. 39. The different humidity stability of the undoped and doped scintillation screens. Photos of changes in $(BTPP)_2MnBr_4$ (undoped) and $(BTPP)_{1.8}(HTPP)_{0.2}MnBr_4$ (doped) under different humidity environments (The red box indicates visible crystallization).

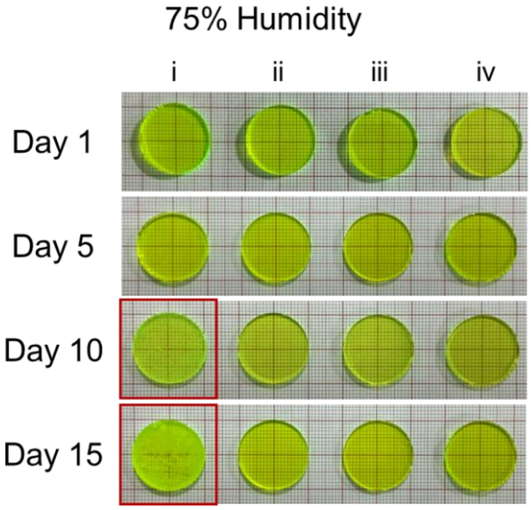

Supplementary Fig. 40. The humidity stability of different transparent scintillation screens. Photos of changes in (i)-(iv) under 75% humidity environments for 15 days ((i): $(BTPP)_2MnBr_4$, (ii): $(BTPP)_{1.8}(HTPP)_{0.2}MnBr_4$, (iii): $(BTPP)_{1.8}(DTPP)_{0.2}MnBr_4$, (iv): $(BTPP)_{1.8}(CTPP)_{0.2}MnBr_4$) (The red box indicates visible crystallization).

We conducted comparative humidity stability tests by exposing different doped glass samples at 25 °C and 75% humidity for 15 days (Supplementary Fig. 40). The results showed that all glass samples doped with long-chain cations, including $HTPP^+$, $DTPP^+$ and $CTPP^+$, remained transparent over the test period, whereas the undoped $(BTPP)_2MnBr_4$ sample exhibited visible crystallization after 10 days. These findings confirm that doping with long-chain cations effectively enhances humidity resistance, and that $DTPP^+$/$CTPP^+$ doped glasses achieve a similar stability improvement to that of $(BTPP)_{1.8}(HTPP)_{0.2}MnBr_4$. However, $DTPP^+$/$CTPP^+$ doped samples were not included in the systematic fast neutron performance tests due to insufficient optical performance.

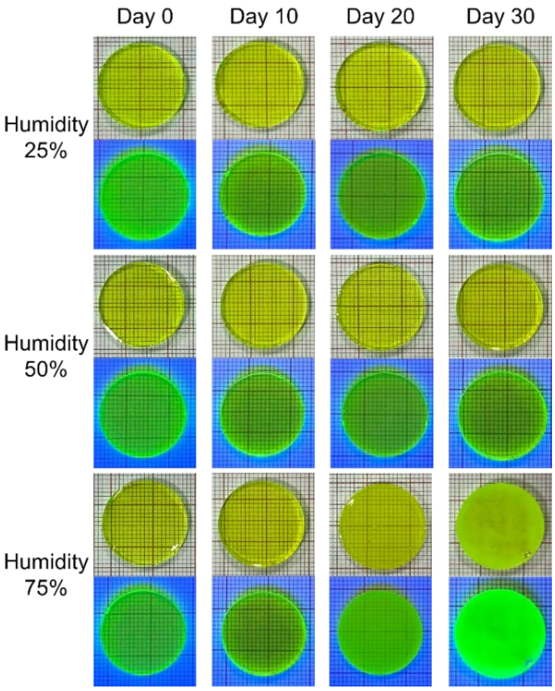

Supplementary Fig. 41. The different humidity stability of $(BTPP)_{1.8}(HTPP)_{0.2}MnBr_4$ for 30 days (up: visible light; bottom: 365 nm UV light).

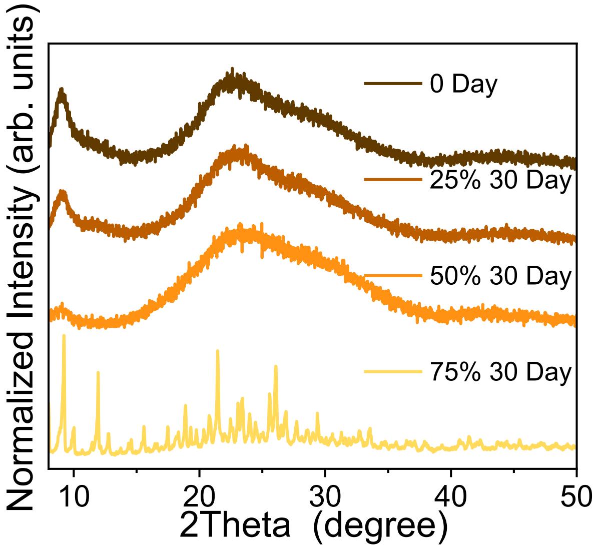

Supplementary Fig. 42. The PXRD of $(BTPP)_{1.8}(HTPP)_{0.2}MnBr_4$ under different humidity for 30 days. The initial PXRD and the PXRD after being stored at 25%, 50% and 75% humidity levels at the same temperature for 30 days of $(BTPP)_{1.8}(HTPP)_{0.2}MnBr_4$ transparent scintillation screen.

To assess the effect of humidity, we tested the transparency retention at 25%, 50% and 75% humidity levels at the same temperature. As shown in Supplementary Fig. 41, the $(BTPP)_{1.8}(HTPP)_{0.2}MnBr_4$ remains transparent at 25% and 50% humidity after 30 days, but becomes opaque at 75% humidity. The PXRD also revealed crystallization peaks under high humidity, indicating a transition from a transparent amorphous state to an opaque crystalline state (Supplementary Fig. 42).

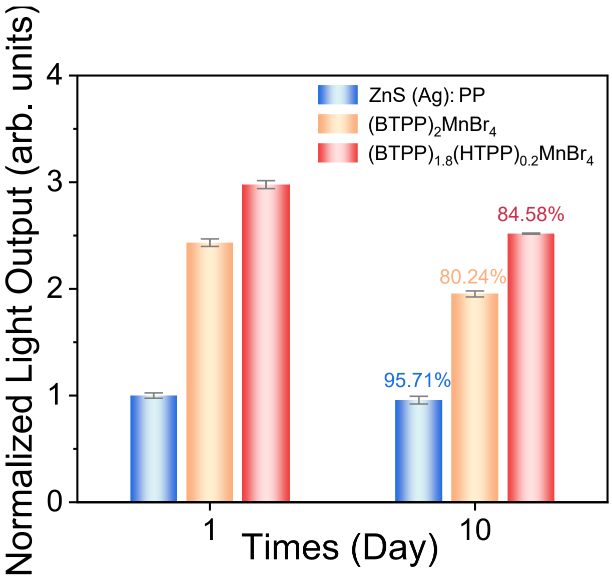

Supplementary Fig. 43. Fast neutron irradiation stability. The stability of ZnS (Ag): PP, $(BTPP)_2MnBr_4$ and $(BTPP)_{1.8}(HTPP)_{0.2}MnBr_4$ scintillation screens under continuous fast neutron irradiation (fast neutron flux: 10^7 n cm^{-2} s^{-1}) (mean ± SD, n = 3).

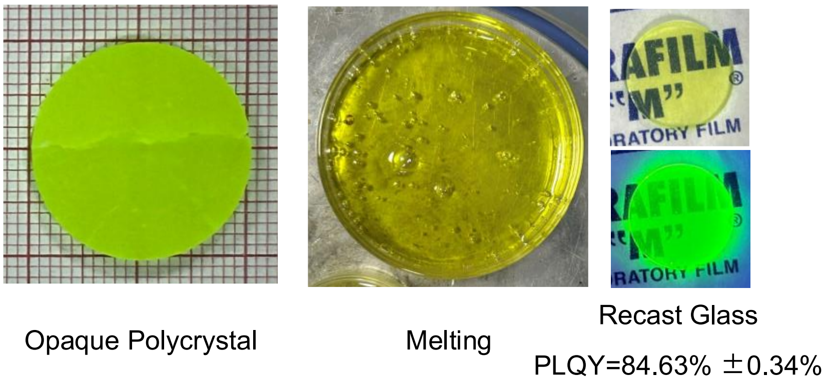

Supplementary Fig. 44. The verification of repeatability of $(BTPP)_{1.8}(HTPP)_{0.2}MnBr_4$. Photos of remolded $(BTPP)_{1.8}(HTPP)_{0.2}MnBr_4$ restored to its original PLQY by re-heating and melting (up: visible light; bottom: 365 nm UV light).

Supplementary References

1. Ma, Y. Y. et al. Solvent-free mechanochemical syntheses of microscale lead-free hybrid manganese halides as efficient green light phosphors. *J. Mater. Chem. C* **9**, 9952-9961 (2021).
2. Mao, L. L., Guo, P. J., Wang, S. X., Cheetham, A. K. & Seshadri, R. Design principles for enhancing photoluminescence quantum yield in hybrid manganese bromides. *J. Am. Chem. Soc.* **142**, 13582-13589 (2020).
3. Shao, W. Y. et al. Highly efficient, flexible, and Eco-friendly manganese(II) halide nanocrystal membrane with low light scattering for high- resolution X-ray imaging. *ACS Appl. Mater. Interfaces* **15**, 932-941 (2023).
4. Wang, X., Zhang, X., Liu, Y. & Zhang, Y. Shape-on-demand synthesis of luminescent $(ETP)_2MnBr_4$ glass scintillator. *Chem. Eng. J.* **483**, 149239 (2024).
5. Xu, Y. K. et al. Organic cation design of manganese halide hybrids glass toward low-temperature integrated efficient, scaling, and reproducible X-ray detector. *Adv. Opt. Mater.* **11**, 2300216 (2023).
6. Luo, J. B. et al. Bisphosphonium cation based metal halide glass scintillators with tunable melting points. *Chem. Sci.* **15**, 16338-16346 (2024).
7. Li, B. H. et al. Zero-dimensional luminescent metal halide hybrids enabling bulk transparent medium as large-area X-ray scintillators. *Adv. Opt. Mater.* **10**, 2102793 (2022).
8. Luo, J. B., Wei, J. H., Zhang, Z. Z., He, Z. L. & Kuang, D. B. A melt-quenched luminescent glass of an organic-inorganic manganese halide as a large-area scintillator for radiation detection. *Angew. Chem. Int. Ed.* **62**, 1-9 (2023).
9. Knyazev, A. et al. Properties of the CsI(Tl) detector elements of the CALIFA detector. *Nucl. Instrum. Methods Phys. Res., Sect. A* **940**, 393-404 (2019).
10. Huajie, W. et al. A geometric unsharpness correction method for neutron photographic image based on improved Richardson–Lucy algorithm. *Nucl. Tech.* **46**, 30-35 (2023).
11. Wu, Y. et al. A preliminary study of the performance for high energy neutron radiography convertor. *Nucl. Tech.* **43**, 070203 (2020).
12. Wang, W. et al. Experimental study of spatial resolution of MCPs for compact high-resolution neutron radiography system. *Nucl. Instrum. Methods Phys. Res., Sect. A* **1050**, 168179 (2023).
13. Malgorzata, M., Bernhard, W., Albert, Z., Christian, G. & Thomas, B. Performance of the commercial PP/ZnS:Cu and PP/ZnS:Ag scintillation screens for fast neutron imaging. *J. Imaging* **3**, 60-72 (2017).
14. Xia, M. et al. Organic–inorganic hybrid perovskite scintillators for mixed field radiation detection. *InfoMat* **4**, e12325 (2022).
15. Zboray, R. et al. High-frame rate imaging of two-phase flow in a thin rectangular channel using fast neutrons. *Appl. Radiat. Isot.* **90**, 122-131 (2014).

16. Oksuz, I., Chuirazzi, W., Martinez, H. P., Cherepy, N. & Cao, L. *Characterization of polyvinyl toluene (PVT) scintillators for fast neutron imaging* (San Diego, CA, 2018).
17. Oksuz, I., Bisbee, M., Hall, J., Cherepy, N. & Cao, L. Quantifying spatial resolution in a fast neutron radiography system. *Nucl. Instrum. Methods Phys. Res., Sect. A* **1027**, 166331 (2022).
18. He, S. et al. Hot exciton-based plastic scintillator engineered for efficient fast neutron detection and imaging. *Adv. Funct. Mater.*, 2503688 (2025).
19. Montanarella, F. et al. Highly concentrated, zwitterionic ligand-capped Mn^{2+}:$CsPb(Br_xCl_{1-x})_3$ nanocrystals as bright scintillators for fast neutron imaging. *ACS Energy Lett.* **6**, 4365-4373 (2021).
20. McCall, K. M. et al. Fast neutron imaging with semiconductor nanocrystal scintillators. *ACS Nano* **14**, 14686-14697 (2020).
21. Shao, W. Y. et al. Synergy of organic and inorganic sites in 2D perovskite for fast neutron and X-ray imaging. *Adv. Funct. Mater.* **33**, 2301767 (2023).
22. Zheng, J. X. et al. Hydrogen-rich 2D halide perovskite scintillators for fast neutron radiography. *J. Am. Chem. Soc.* **143**, 21302-21311 (2021).
23. Yang, B. et al. Inch-sized 2D perovskite single-crystal scintillators for high-resolution neutron and X-ray imaging. *InfoMat*, e12648 (2024).
24. Yan, W. et al. Organic–inorganic hybrid perovskite scintillator for neutron and γ-ray detection. *ACS Appl. Opt. Mater.* **1**, 1856-1861 (2023).
25. Lukosi, E. et al. First evaluation of fast neutron imaging with $LiInSe_2$ semiconductors. *Nucl. Instrum. Methods Phys. Res., Sect. A* **976**, 164254 (2020).
