# Supplementary material for: A transparent hybrid metal halide glassy scintillation screen for high-resolution fast neutron radiography
Source: Nat Commun. 2025 Jul 5;16:6215. doi: 10.1038/s41467-025-61503-9 (PMC12228732; doi:10.1038/s41467-025-61503-9)
Supplement: Supplementary file 2 — Description of Additional Supplementary Files [file 41467_2025_61503_MOESM2_ESM.pdf]

Description of Additional Supplementary Files

File Name: Supplementary Data 1

Description: Crystallographic data of $(BTPP)_2MnBr_4$ ($(C_{22}H_{24}P)_2MnBr_4$) single crystal (CCDC number: 2456170).

File Name: Supplementary Data 2

Description: Crystallographic data of $(BTPP)_2MnBr_4$ molten state from theoretical calculations.

File Name: Supplementary Data 3

Description: Crystallographic data of $(BTPP)_2MnBr_4$ polycrystalline state from theoretical calculations.

File Name: Supplementary Data 4

Description: Crystallographic data of $(BTPP)_2MnBr_4$ glassy state from theoretical calculations.
